# The Association Between Metabolic Dysfunction‐Associated Steatotic Liver Disease and Change in Liver Stiffness in Patients With Chronic Hepatitis B

**DOI:** 10.1111/liv.70042

**Published:** 2025-02-25

**Authors:** Lesley A. Patmore, Kirsi van Eekhout, Özgür M. Koc, Robert J. de Knegt, Harry L. A. Janssen, Willem P. Brouwer, Matthijs Kramer, Pieter Honkoop, Joep de Bruijne, Greet J. Boland, Douwe F. Postma, Hans Blokzijl, Robert A. de Man, R. Bart Takkenberg, Milan J. Sonneveld

**Affiliations:** ^1^ Department of Gastroenterology and Hepatology, Erasmus MC University Medical Center Rotterdam the Netherlands; ^2^ Department of Gastroenterology and Hepatology Amsterdam UMC Amsterdam the Netherlands; ^3^ Department of Gastroenterology and Hepatology Maastricht University Medical Center Maastricht the Netherlands; ^4^ Toronto Center for Liver Disease, Toronto General Hospital University of Toronto Toronto Ontario Canada; ^5^ Department of Pathology, GROW‐School for Oncology and Developmental Biology Maastricht University Medical Center+ Maastricht the Netherlands; ^6^ Department of Gastroenterology and Hepatology Albert Schweitzer Hospital Dordrecht the Netherlands; ^7^ Department of Gastroenterology and Hepatology University Medical Center Utrecht Utrecht the Netherlands; ^8^ Department of Medical Microbiology University Medical Center Utrecht Utrecht the Netherlands; ^9^ Department of Internal Medicine and Infectious Diseases University Medical Center Groningen Groningen the Netherlands; ^10^ Department of Gastroenterology and Hepatology University Medical Center Groningen Groningen the Netherlands

**Keywords:** liver stiffness measurement, MAFLD, MASLD, metabolic comorbidities, NAFLD

## Abstract

**Background and Aims:**

Metabolic dysfunction‐associated steatotic liver disease (MASLD) is associated with an increased risk of liver‐related events in patients with chronic hepatitis B (CHB), possibly by accelerating fibrosis progression. Therefore, we studied the influence of MASLD on liver stiffness measurement (LSM) kinetics in CHB patients.

**Methods:**

We conducted a multicenter retrospective cohort study of CHB patients with at least two LSM with FibroScan. We studied the absolute change in LSM and the change in LSM stage from the first LSM to the most recent LSM among CHB patients with MASLD compared to patients without MASLD.

**Results:**

We analysed 1055 CHB patients; 259 (28.0%) had MASLD. Patients with MASLD had a higher first and last LSM (6.1 vs. 5.2 kPa and 5.6 vs. 4.7 kPa, *p* < 0.001), were significantly less likely to achieve a decrease in LSM stage (52.8% vs. 74% *p* < 0.001) and were more likely to experience an increase in LSM stage (19.3% vs. 13.6%, *p* = 0.035) during follow‐up. 417 (39.5%) patients initiated antiviral therapy (AVT) which was associated with a decline in LSM (*p* < 0.001). However, patients with MASLD who were treated were less likely to decrease in LSM stage (52.4% vs. 77.0%, *p* < 0.001) and were more likely to experience an increase in LSM stage (23.5% vs. 12.8%, *p* = 0.021) despite AVT.

**Conclusion:**

Presence of MASLD was independently associated with higher LSM in untreated CHB patients and with less decline in LSM after initiation of AVT. Furthermore, CHB patients with MASLD were more likely to experience an increase in LSM despite AVT.

**Abbreviations**
AASLD: American Association for the Study of Liver Disease
ALT: alanine aminotransferase
aOR: adjusted odds ratio
BMI: body mass index
CHB: chronic hepatitis B
CI: confidence interval
EASL: European Association for the Study of the Liver
HBeAg: hepatitis B e antigen
HBsAg: hepatitis B surface antigen
HBV: hepatitis b virus
HBV DNA: hepatitis b virus deoxyribonucleic acid
HCC: hepatocellular carcinoma
HCV: hepatitis C virus
HDV: hepatitis D virus
HIV: human immunodeficiency virus
IQR: interquartile range
LSM: liver stiffness measurement
MAFLD: metabolic‐ddysfunction associated fatty liver disease
MASLD: metabolic‐ddysfunction associated steatotic liver disease
NAFLD: non‐aAlcoholic fatty liver disease
NUC: nucleo(s)tide analogue
ULN: upper limit of normal

**Summary**

- Chronic hepatitis B patients with MASLD have a baseline higher liver stiffness compared to patients without MASLD.
- Besides, the decline in liver stiffness after initiation of antiviral therapy is less and, in some cases, even increases among patients with MASLD

## 1 Introduction

Chronic hepatitis B (CHB) is a global healthcare problem that currently affects approximately 254 million people worldwide [1]. CHB causes almost 800,000 deaths annually as a consequence of liver cirrhosis and hepatocellular carcinoma (HCC). Viral suppression with nucleo(s)tide analogues (NUCs) reduces liver inflammation and may reduce fibrosis and HCC risk [2, 3]. However, in some patients, fibrosis continues to progress and HCC still occurs, so regular monitoring of these patients is crucial [4].

The persistent risk of fibrosis progression and adverse liver‐related outcomes may be partially attributable to the presence of co‐existing liver disease such as metabolic dysfunction‐associated steatotic liver disease (MASLD) formerly known as non‐alcoholic fatty liver disease (NAFLD) or metabolic dysfunction‐associated fatty liver disease (MAFLD) [5, 6]. Recent studies report that the presence of metabolic dysfunction in combination with steatotic liver disease is associated with an excess risk of adverse clinical outcomes in patients with CHB [7, 8]. These findings are supported by another study reporting a higher risk of liver‐related events in the presence of one or more metabolic risk factors such as diabetes mellitus, hypertension, dyslipidaemia and overweight [9]. These associations could be attributable to an increased risk of fibrosis progression among CHB patients co‐affected by MASLD [10]. This hypothesis is supported by a study showing higher rates of significant fibrosis in patients with CHB and steatosis, but data on the association between MASLD and fibrosis kinetics during follow‐up are currently lacking [11]. Liver stiffness assessment is a non‐invasive method to assess the stage of liver disease in patients with CHB and can potentially be used as a non‐invasive tool to monitor changes in liver disease severity over time [12]. The aim of the current study was to investigate the influence of the presence of MASLD on LSM kinetics in patients with CHB.

## 2 Methods

### 2.1 Study Cohort

We conducted a multicenter retrospective cohort study of mono‐infected CHB patients who underwent at least two LSMs with vibration‐controlled transient elastography (FibroScan, Echosens) at least 6 months apart. Patients were included if they were not on antiviral therapy at the time of the first LSM and had a serum alanine aminotransferase (ALT) < 5× upper limit of normal (ULN). Patients were excluded in case of (past) viral co‐infections (HDV, HCV and HIV), use of medication potentially causing steatosis (e.g., prednisone), documented alcohol misuse or presence or development of other known chronic liver diseases (documented alcoholic liver disease, Wilson's disease, primary biliary cholangitis, primary sclerosing cholangitis, auto‐immune hepatitis and hemochromatosis).

### 2.2 Data Collection and Study Definitions

Baseline and follow‐up data were collected on demographics, antiviral treatment, liver biochemistry and virology. MASLD was defined as the presence of hepatic steatosis (based on histology, controlled attenuation parameter ≥ 248 dB/m and/or ultrasound) in combination with metabolic dysfunction (diabetes, overweight, dyslipidaemia or hypertension) [6, 13]. The presence of metabolic dysfunction was assessed by chart review: (1) diabetes mellitus, which was based on medical history or use of antidiabetic medication, (2) hypertension, which was based on the medical history or use of antihypertensives, (3) dyslipidaemia, which was based on medical history or statin use and (4) overweight, defined as BMI ≥ 25 kg/m^2^ for non‐Asians and ≥ 23 kg/m^2^ for Asians [14].

All initial LSMs were conducted on untreated patients, and the initiation of subsequent antiviral therapy was at the discretion of the treating physician, with indications based on the EASL guidelines [15]. In the case of the initiation of antiviral therapy during follow‐up, patients were censored and transitioned to the treated group. HBV DNA below the level of detection was defined as HBV DNA < 80 IU/mL.

Fibroscan (Echosens, Paris, France) examination was performed in each center by nurses or physicians trained and certified by the manufacturer and using either M and XL probes as per manufacturers recommendations. All patients were asked to fast at least 3 h before the examination. Only examinations with at least 10 valid individual measurements and acceptable variances (< 30%) were deemed valid. LSM was categorised in LSM stages according to the European Association for study of the liver (EASL) guidelines; LSM < 6 kPa was defined as ‘minimal fibrosis’, LSM > 9 kPa was defined as ‘advanced fibrosis’ and LSM 6–9 kPa was defined as a ‘grey area’ [16].

### 2.3 Study Outcome

The primary outcomes were the absolute change in LSM and the change in LSM stage from the first LSM to the most recent LSM among CHB patients co‐affected by MASLD compared to patients without MASLD. An increase in LSM stage was defined as an increase of at least one stage among patients with a first LSM < 9 kPa. A decrease in LSM stage was defined as a decrease in LSM of at least one stage among patients with a first LSM > 6 kPa.

Secondary analyses focused on the association between the presence of MASLD and change in LSM in patients with and without antiviral therapy.

### 2.4 Statistical Analysis

Cohort characteristics were described as counts with percentages (%) for categorical variables, as means with the standard deviation (SD) for normally distributed continuous variables and as medians with the interquartile range (IQR) for non‐normally distributed continuous variables. Differences between sub‐groups were analysed using the independent sample *t*‐test for normally distributed continuous data, the Mann–Whitney *U* test for non‐normally distributed continuous data and the Chi‐square for categorical data. Baseline was set on the date of the first available LSM.

Differences between first and last LSM and absolute LSM changes were compared using the Mann–Whitney *U* test, and the changes in LSM stage over time were compared using the chi‐square test. Analyses were performed in the overall population and stratified by antiviral treatment. The association between the presence of MASLD and LSM values was explored using multivariable linear regression analyses. Finally, we performed a logistic regression adjusting for age, sex, ALT, antiviral therapy, baseline LSM and follow‐up time to explore the association between the presence of MASLD and LSM > 9 kPa at last follow‐up. Differences were considered statistically significant when *p* < 0.05. For statistical data analysis, IBM SPSS for Windows version 25.0 (SPSS Inc., Chicago, IL, USA) was used.

### 2.5 Ethics

This study was conducted in accordance with the guidelines of the Declaration of Helsinki and the principles of Good Clinical Practice. The requirement for informed consent was waived, and the individual institutional review boards gave necessary approval. The study protocol was reviewed by the Erasmus MC Medical Ethical Committee (MEC‐2021‐0919).

## 3 Results

### 3.1 Study Cohort

We analysed 1055 CHB patients with a median age of 39 years (IQR 31–48) at baseline. 53.2% were male, and patients were predominantly Caucasian (34.5%) or Asian (34.4%). 239 (22.7%) were HBeAg positive, and median HBV DNA log_10_ was 3.4 UI/mL (IQR 2.2–5.1) at baseline. Genotype determination was performed in only 584 (55.3%) patients. The most common HBV genotype was D (22.2%), followed by genotype C (16.6%), genotype A (15.6%) and B (14.2%). Patients with MASLD were older, more often HBeAg negative and had slightly lower HBV DNA levels when compared to subjects without MASLD.

In the overall cohort, steatosis was confirmed in 100 patients through biopsy, 102 patients had CAP measurements > 248 dB/m and 248 patients had ultrasound findings indicative of steatosis. A total of 390 patients were diagnosed with hepatic steatosis based on (a combination of) these findings. The majority (54.7%) had one or more metabolic comorbidities; 507 (48.1%) patients were overweight, 141 (13.4%) had hypertension, 122 (11.6%) had dyslipidaemia and 65 (6.2%) had diabetes. Two hundred and ninety‐five patients (28.0%) met the MASLD criteria. 417 (39.5%) patients started antiviral therapy with NUC during follow‐up. The baseline characteristics for the overall cohort and for patients with and without MASLD are described in Table 1.

**TABLE 1:** Baseline characteristics. Data are presented as mean (SD) for normally distributed variables, as median (IQR) for non‐normally distributed data and as *n*(%) for categorical variables.

	Total (*n* = 1055)	Non‐MASLD (*n* = 760)	MASLD (*n* = 295)	*p*
Age, years	39 (31–48)	37 (29–46)	45 (36–54)	< 0.001
Male sex, *n* (%)	561 (53.2)	363 (47.8)	198 (67.1)	< 0.001
Ethnicity, *n* (%)				
Caucasian	364 (34.5)	240 (31.6)	124 (42.0)	0.032
Asian	363 (34.4)	276 (36.3)	87 (29.5)	
Sub‐Saharan African	240 (22.7)	182 (23.9)	58 (19.7)	
Other	88 (8.3)	62 (8.2)	26 (8.8)	
ALT, IU/mL	34 (23–50)	31 (22–47)	40 (27–57)	< 0.001
Albumin, g/L	44 (4.3)	44 (4.2)	45 (4.5)	0.006
Platelet count, ×10^9^/L	222 (187–258)	220 (187–255)	224 (190–272)	0.072
HBV DNA log_10_ IU/mL	3.4 (2.2–5.1)	3.6 (2.4–5.5)	3.1 (1.8–4.2)	< 0.001
HBV DNA > 2000 IU/mL, *n* (%)	805 (79.2)	608 (81.4)	197 (73.2)	0.005
HBeAg positive, *n* (%)	239 (22.7)	204 (26.8)	35 (11.9)	< 0.001
Genotype, *n* (%)	584 (55.3)	355 (46.7)	110 (37.2)	
A	91 (15.6)	71 (20.0)	20 (18.2)	0.006
B	83 (14.2)	68 (19.2)	15 (5.0)	
C	97 (16.6)	79 (22.2)	18 (16.3)	
D	130 (22.2)	86 (24.2)	44 (40.0)	
E	60 (10.3)	47 (13.2)	13 (11.2)	
Other	4 (0.7)	4 (1.1)	0 (0)	
Antiviral therapy with NUC, *n* (%)	417 (39.5)	291 (38.3)	126 (42.7)	
Tenofovir	226 (54.2)	164 (56.4)	62 (49.2)	0.187
Entecavir	178 (42.7)	117 (40.2)	61 (48.4)	
Other	13 (1.2)	10 (1.3)	3 (2.4)	
Steatosis, *n* (%)	390 (37.0)	95 (12.5)	295 (100.0)	< 0.001
Overweight, *n* (%)	507 (48.1)	237 (31.2)	270 (91.5)	< 0.001
Diabetes, *n* (%)	65 (6.2)	17 (2.2)	48 (16.3)	< 0.001
Hypertension, *n* (%)	141 (13.4)	56 (7.4)	85 (28.8)	< 0.001
Dyslipidaemia, *n* (%)	122 (11.5)	41 (5.4)	81 (27.5)	< 0.001

### 3.2 Influence of MASLD on LSM Change During Follow‐Up

The median LSM at baseline was 5.4 kPa (IQR 4.4–7.3). Patients co‐affected by MASLD had a significantly higher LSM at baseline compared to patients without MASLD (6.1 kPa [IQR 4.9–8.3] vs. 5.2 kPa [IQR 4.2–6.7], *p* < 0.001). Median follow‐up time between the first and last LSM was 4.7 years (IQR 2.8–7.4); there was no difference between follow‐up time for patients with or without MASLD (4.4 years [IQR 2.6–7.0] vs. 4.7 years [IQR 2.8–7.5], *p* = 0.175).

The median LSM at last follow‐up was 4.9 kPa (IQR 4.0–6.2); patients co‐affected by MASLD had a significantly higher follow‐up LSM compared to patients without MASLD (5.6 kPa [IQR 5.6–7.4] vs. 4.7 kPa [IQR 3.9–5.8], *p* < 0.001).

**FIGURE 1:**
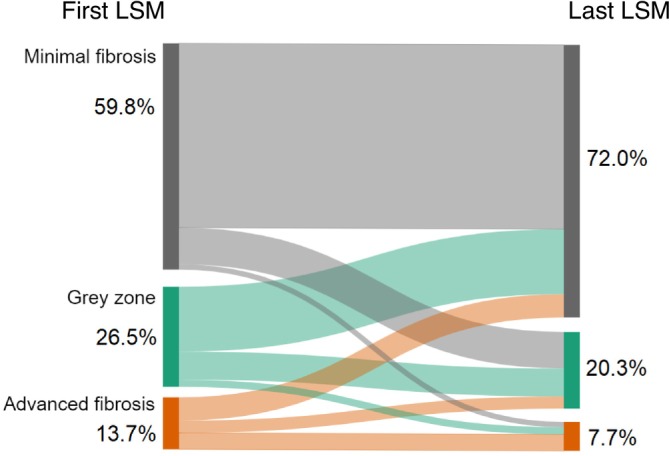
Sankey plot illustrating the transition of patients from the first LSM stage to the last LSM stage in the overall cohort.

**FIGURE 2:**
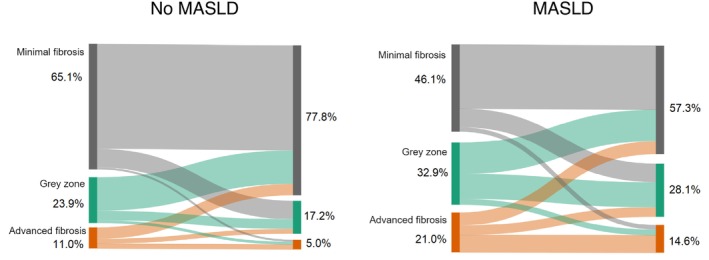
Sankey plot illustrating the transition of patients from the first LSM stage to the last LSM stage, stratified by the presence of MASLD.

Based on the baseline LSM, 631 patients (59.8%) were classified as having minimal fibrosis and 145 (13.7%) as having advanced fibrosis. Patients co‐affected by MASLD were more likely to have advanced fibrosis (21.1% vs. 10.9%, *p* < 0.001). In the overall cohort, the median difference between the first and last LSM was −0.5 kPa (IQR −1.9—0.7). The majority of the patients (60.5%) remained in the same LSM stage. Only 2.4% (*n* = 15) of the patients with minimal fibrosis progressed to advanced fibrosis and 44.8% (*n* = 65) of the patients with advanced fibrosis decreased to minimal fibrosis based on the last LSM. The change in liver stiffness of the overall cohort and stratified by the presence of MASLD, is depicted in Figures 1 and 2.

Of the 424 patients with baseline LSM > 6 kPa, 66% had a decrease in LSM stage.

Patients with MASLD were significantly less likely to achieve a decrease in LSM stage compared to patients without MASLD (52.8% vs. 74%, *p* < 0.001). Of the 907 patients with baseline LSM < 9 kPa, 15% had an increase in LSM stage. Patients with MASLD were significantly more likely to experience an increase in LSM stage compared to patients without MASLD (19.3% vs. 13.6% *p* = 0.035). All LSM kinetics are summarised in Table 2.

**TABLE 2:** LSM kinetics of the overall cohort and stratified by the presence of MASLD.

	Overall cohort (*n* = 1055)	No MASLD (*n* = 760)	MASLD (*n* = 295)	*p*
*First LSM*				
Median LSM, kPa (IQR)	5.4 (4.4–7.3)	5.2 (4.2–6.7)	6.1 (4.9–8.3)	< 0.001
LSM category, *n* (%)				
Minimal fibrosis	631 (59.8)	495 (65.1)	136 (46.1)	< 0.001
Grey zone	279 (26.4)	182 (24.0)	97 (32.9)	
Advanced fibrosis	145 (13.7)	83 (10.9)	62 (21.0)	
*Last LSM*				
Median LSM, kPa (IQR)	4.9 (4.0–6.2)	4.7 (3.9–5.8)	5.6 (4.5–7.4)	< 0.001
LSM category, *n* (%)				
Minimal fibrosis	760 (72.0)	591 (77.8)	169 (57.3)	< 0.001
Grey zone	214 (20.3)	131 (17.2)	83 (28.1)	
Advanced fibrosis	81 (7.7)	38 (5.0)	43 (14.6)	

### 3.3 Influence of Antiviral Therapy

417 (39.5%) patients initiated antiviral therapy during follow‐up, of whom 126 (30.2%) had MASLD. There was no significant difference in the proportion of patients with MASLD on antiviral therapy and the proportion of patients without MASLD on antiviral therapy (42.7% vs. 38.2%, *p* = 0.187).

Initiation of antiviral therapy was associated with a decline in LSM versus no treatment (−1.2 kPa vs. −0.2 kPa, *p* < 0.001). Patients with MASLD experienced a smaller decrease in LSM compared to patients without MASLD (−0.6 kPa [IQR −3.2 to 1.3] vs. −1.5 kPa [IQR −3.4 to −0.1], *p* = 0.02). Among treated patients with LSM > 9 kPa at baseline, patients with MASLD also had significantly higher LSM at follow‐up compared to patients without MASLD (9.0 kPa [IQR 4.8–13.5] vs. 5.8 kPa [IQR 4.7–8.1], *p* = 0.016).

Among the treated patients with baseline LSM > 6 kPa, 68.4% had a decrease in LSM stage. Treated patients with MASLD were less likely to decrease in LSM stage compared to patients without MASLD (52.4% vs. 77.0%, *p* < 0.001). Among the treated patients with baseline LSM < 9 kPa, 15.5% had an increase in LSM stage. Treated patients with MASLD were more likely to increase in LSM stage compared to treated patients without MASLD (23.5% vs. 12.8%, *p* = 0.021).

### 3.4 MASLD Is Independently Associated With Higher LSM at Baseline and at Last Follow‐Up

In linear regression adjusting for age, sex, ALT and HBV DNA log_10_, we found that the presence of MASLD was significantly associated with a higher first LSM (*p* = 0.006, Table S1). Additionally adjusting for the use of antiviral therapy, first LSM and follow‐up time, we also found that MASLD was significantly associated with a higher last LSM (*p* = 0.019, Table S2).

Multivariable logistic regression showed that higher age (adjusted odds ratio [aOR] 1.035, 95% CI 1.013–1.057, *p* = 0.002), higher ALT levels (aOR 1.007, 95% CI 1.003–1.010, *p* < 0.001), higher first LSM (aOR 1.259, 95% CI 1.187–1.335, *p* < 0.001), shorter follow‐up time (aOR 0.869, 95% CI 0.792–0.953, *p* = 0.003) and MASLD (aOR 2.294, 95% CI 1.311–4.015, *p* = 0.004) were significantly associated with a higher risk of LSM > 9 kPa at follow‐up (Table 3).

**TABLE 3:** Association between MASLD and risk of last LSM > 9 kPa using multivariable logistic regression analysis.

	aOR	95% CI	*p*
Age, years	1.035	1.013–1.057	0.002
Male sex	1.454	0.783–2.701	0.236
ALT, U/L	1.007	1.003–1.010	< 0.001
Use of antiviral therapy	1.494	0.813–2.748	0.196
First LSM, kPa	1.259	1.187–1.335	< 0.001
Time between first and last LSM, years	0.869	0.792–0.953	0.003
Presence of MASLD	2.294	1.311–4.015	0.004

## 4 Discussion

In this multicenter study involving 1055 CHB patients, we found that the presence of MASLD was independently associated with a higher liver stiffness in untreated patients and with less decline in LSM after initiation of antiviral therapy. Furthermore, CHB patients with MASLD were more likely to experience an increase in LSM even if antiviral therapy was initiated.

The increasing prevalence of metabolic syndrome and hepatic steatosis has brought attention to their potential interactions with chronic viral hepatitis. Previous studies have demonstrated that hepatic steatosis correlates with liver fibrosis and persistent severe steatosis appears to accelerate fibrosis progression in patients with CHB [11]. Moreover, there is an association between metabolic syndrome, fatty liver disease and cirrhosis in CHB patients [17, 18]. Recently, a multi‐stakeholder consensus working group has proposed the term MASLD for steatotic liver disease in the presence of cardiometabolic risk factors [6].

In the current study, patients co‐affected by MASLD had a significantly higher LSM and were more likely to have LSM‐based advanced fibrosis at study enrolment compared to patients without MASLD, a finding that further underscores the importance of MASLD as a risk factor for developing liver fibrosis in patients with CHB. Patients with MASLD were older, more often HBeAg negative and had slightly lower HBV DNA levels when compared to subjects without MASLD. This is mainly due to the association of age with both MASLD and the higher prevalence of HBeAg negative CHB (which is associated with lower HBV DNA levels). However, as can be judged from Table 1, mean HBV DNA levels were still over 3 log IU/mL for patients with MASLD and 73.2% had HBV DNA levels > 2000 IU/mL. These patients therefore have significant HBV replication and are at risk of HBV related disease progression. To confirm our results, we performed multiple multivariable regression analyses in which we adjusted for these factors and found that the presence of MASLD independently increased the risk of higher LSM.

Interestingly, antiviral therapy was associated with a marked reduction in LSM in the majority of patients. This decline likely reflects improvements in fibrosis as well as inflammation that is typically observed with prolonged suppression of viral replication [3]. Importantly, we also observed that the presence of MASLD attenuated the positive effects of antiviral therapy on LSM and the presence of MASLD was even associated with a significant risk of LSM increase despite antiviral therapy. The correlation between LSM and histological fibrosis grade during antiviral therapy is still unclear, given the lack of biopsy‐controlled studies. However, these findings make it highly suggestive that the presence of MASLD is associated with less improvement or even worsening, of liver fibrosis, despite antiviral therapy. These findings are very relevant in the light of recent observations that the presence of cardiometabolic risk factors and associated steatotic liver disease are risk factors for liver‐related events in patients with CHB [7, 9, 10]. In a previous study from our research group, we found that the 5‐year cumulative incidence of liver‐related events in CHB patients with cirrhosis and one or more metabolic comorbidities was 13%–22% compared to 6%–11% in patients without cirrhosis with metabolic comorbidities [9]. To investigate whether the presence of steatosis in combination with another metabolic comorbidity has an additive effect for fibrosis progression, we performed a sensitivity analysis. Multivariable logistic regression showed that the concomitant presence of metabolic comorbidities and steatosis (i.e., MASLD) was associated with a higher risk of LSM > 9 kPa at follow‐up compared to metabolic comorbidities alone (Table S3). A potentially relevant question is which comorbidity has the most influence on LSM kinetics. We attempted to study this by incorporating the individual comorbidities in a multivariable model (Table S4). However, interpretation of these estimates is limited by the fact that only a few patients had only one comorbidity other than overweight.

Early recognition and management of metabolic syndrome in CHB patients could yield long‐term benefits. Lifestyle modifications such as increased physical activity, weight loss and dietary changes can positively impact metabolic syndrome components. Furthermore, preliminary data suggest that adequate control of DM can reduce the risk of liver‐related events in CHB patients co‐affected by DM [19]. Additionally, the presence of metabolic syndrome might prompt a reconsideration of CHB treatment strategies due to the elevated cirrhosis risk, possibly warranting a lower threshold for initiating antiviral therapy.

Even though our present study had the strength of a large sample size, there were some limitations. First, data were collected retrospectively, and detailed information on the degree of alcohol consumption over time was not always documented. Second, we assessed metabolic comorbidities at baseline, and there was not always data on the development of metabolic comorbidities during follow‐up. However, this would lead to underdiagnosis of MASLD and would only result in a bias towards finding no association, and it is therefore unlikely to have influenced the outcomes of this study. Third, since most (on‐treatment) CHB patients are stable and asymptomatic, we relied on LSM for fibrosis assessment instead of liver biopsies, which is the golden histological standard. LSM correlates well with fibrosis stage in treatment‐naïve CHB patients, but its correlation with fibrosis changes during treatment is yet uncertain [12]. Additional studies, including liver biopsy, are required to confirm whether an increase in LSM reflects fibrosis progression after initiation of antiviral therapy in patients co‐affected by MASLD. The same applies to CAP measurement, where the optimal cut‐off to assess the presence of steatosis remains a matter of debate. For our study, we used the cut‐off value of > 248 dB/m as used and recommended by the manufacturer [13]. In the EASL guideline for non‐invasive tests recommends a higher cut‐off of > 275 dB/m is recommended for the diagnosis of NAFLD/NASH [20] We performed a sensitivity analysis and found consistent results, which are summarised in Tables S5–S8.

In conclusion, the current study showed that untreated CHB patients co‐affected by MASLD had a higher LSM at baseline and were more likely to experience an increase in LSM during follow‐up. While the initiation of antiviral therapy was associated with a decrease in LSM in the overall study population, such a decrease was significantly less frequently observed among CHB patients with MASLD. Moreover, CHB patients with MASLD were even more likely to increase in LSM despite the initiation of antiviral therapy.

## Author Contributions

Collection of data: L.A.P., K.v.E. Study design, writing of the manuscript and approval of final version: L.A.P. and M.J.S. Critical review of the manuscript and approval of final version: K.v.E., Ö.M.K., R.J.d.K., H.L.A.J., W.P.B., M.K., P.H., J.d.B., G.J.B., D.F.P., H.B., R.A.d.M., and R.B.T.

## Ethics Statement

This study was conducted in accordance with the guidelines of the Declaration of Helsinki and the principles of Good Clinical Practice. The study protocol was reviewed by the Erasmus MC Medical Ethical Committee (MEC‐2021‐0919).

## Consent

The requirement for informed consent was waived, and the individual institutional review boards gave necessary approval.

## Conflicts of Interest

L.A.P. received a research grant from Gilead Sciences. R.J.d.K. received a grant from Echosens, Gilead Sciences, GlaxoSmithKline, Inventiva, Janssen‐Cialg. Consultancy and/or speakers fee from Abbvie, Gilead Sciences, Janssen‐Cilag, Schallware. H.L.A.J. received grants from: Gilead Sciences, GlaxoSmithKline, Janssen, Roche, Vir Biotechnology. Consultant for: Aligos, Gilead Sciences, GlaxoSmithKline, Grifols, Roche, Vir Biotechnology Inc., Precision Biosciences. W.P.B. received a speakers fee from Eli Lilly. Is on the advisory board of Novo Nordisk. Participated in trials from Inventive pharma, Boehringer Ingelheim and 89BIO. M.K. received a speakers fee from Norgine. J.d.B. research support from Terumo. D.F.P. Member of DSMB of the COBRA‐trial (Very short‐course versus standard course antibiotic therapy in patients with acute ChOlangitis after adequate endoscopic BiliaRy drAinage [COBRA trial]; consultancy fees from Gilead [payed to institution]). R.A.d.M. research support from Roche. M.J.S. Speakers fees and research support from Gilead, Roche, Fujirebio and consultancy fees from Gilead and Albireo. K.v.E., Ö.M.K., P.H., G.J.B., H.B., and R.B.T. none.

## Supporting information


Data S1.

## Data Availability

The data that support these findings are not publicly available, since they are subject to (inter)national data protection laws to ensure data privacy of the study participants. The data can therefore not be shared.
