# Supplementary material for: The Association Between Metabolic Dysfunction‐Associated Steatotic Liver Disease and Change in Liver Stiffness in Patients With Chronic Hepatitis B
Source: Liver Int. 2025 Feb 25;45(3):e70042. doi: 10.1111/liv.70042 (PMC11855902; doi:10.1111/liv.70042)
Supplement: Supplementary file 1 — Data S1. [file LIV-45-0-s001.docx]

**SUPPLEMENTARY**

**Supplementary table 1. Association between MASLD and first LSM using multivariable linear regression**

|  | **Coefficient** | **p-value** |
| --- | --- | --- |
| **Age, years** | 0.050 | <0.001 |
| **Male sex** | 1.000 | <0.001 |
| **ALT, U/L** | 0.035 | <0.001 |
| **HBV DNA log_10_, IU/mL** | 0.114 | 0.033 |
| **Presence of MASLD** | 0.743 | 0.006 |

**Supplementary table 2. Association between MASLD and last LSM using multivariable linear regression**

|  | **Coefficient** | **p-value** |
| --- | --- | --- |
| **Age, years** | 0.020 | 0.050 |
| **Male sex** | 0.245 | 0.352 |
| **ALT, U/L** | 0.014 | <0.001 |
| **Use of antiviral therapy** | 0.074 | 0.793 |
| **First LSM, kPa** | 0.463 | <0.001 |
| **Time between first and last LSM, years** | -0.114 | 0.004 |
| **Presence of MASLD** | 0.692 | 0.019 |

**Supplementary table 3. Association between presence of metabolic comorbidities with or without concomitant steatosis (MASLD) with LSM >9 kPa at follow-up using multivariable logistic regression analysis**

|  | **aOR** | **95% CI** | **p-value** |
| --- | --- | --- | --- |
| **Age, years** | 1.033 | 1.011 – 1.056 | 0.003 |
| **Male sex** | 1.444 | 0.777 – 2.681 | 0.245 |
| **ALT, U/L** | 1.007 | 1.003 – 1.010 | <0.001 |
| **Use of antiviral therapy** | 1.514 | 0.822 – 2.788 | 0.183 |
| **First LSM, kPa** | 1.258 | 1.186 – 1.334 | <0.001 |
| **Time between first and last LSM, years** | 0.870 | 0.793 – 0.955 | 0.003 |
| **Presence of**   - **No metabolic comorbidities** - **≥1 metabolic comorbidities alone** - **≥1 metabolic comorbidities with steatosis** | *Reference*  1.468  2.768 | -  0.663 – 3.253  1.381 – 5.548 | <0.001  0.344  0.004 |

**Supplementary table 4. Association between the individual metabolic comorbidities (diabetes, hypertension, dyslipidemia and overweight) and last LSM using multivariable linear regression.**

|  | **Coefficient** | **p-value** |
| --- | --- | --- |
| **Age, years** | 0.014 | 0.237 |
| **Male sex** | 0.953 | <0.001 |
| **Use of antiviral therapy** | 0.820 | 0.003 |
| **Steatosis** | 0.444 | 0.144 |
| **Overweight** | 0.757 | 0.008 |
| **Diabetes** | 1.833 | 0.003 |
| **Hypertension** | 1.301 | 0.005 |
| **Dyslipidemia** | 0.407 | 0.383 |

**Supplementary table 5. LSM kinetics of the overall cohort and stratified by the presence of MASLD based on higher cut-off CAP of >275 dB/m.**

|  | **No MASLD**  **(n=793)** | **MASLD**  **(n=262)** | **p-value** |
| --- | --- | --- | --- |
| **First LSM** |  |  |  |
| Median LSM, kPa (IQR) | 5.2 (4.2 – 6.7) | 6.2 (5.0 – 8.7) | <0.001 |
| LSM category, n (%)   - Minimal fibrosis - Grey zone - Advanced fibrosis | 515 (64.9)  194 (24.5)  84 (10.6) | 116 (44.3)  85 (32.4)  61 (23.3) | <0.001 |
| **Last LSM** |  |  |  |
| Median LSM, kPa (IQR) | 4.7 (3.9 – 5.8) | 5.8 (4.5 – 7.5) | <0.001 |
| LSM category, n (%)   - Minimal fibrosis - Grey zone - Advanced fibrosis | 614 (77.4)  140 (17.7)  39 (4.9) | 146 (55.7)  74 (28.2)  42 (16.0) | <0.001 |

|  | **No MASLD** | **MASLD** | **p-value** |
| --- | --- | --- | --- |
| Decrease in LSM stage | 73% | 52.7% | <0.001 |
| Increase in LSM stage | 13.5% | 20.4% | 0.016 |
|  | **No MASLD on AVT** | **MASLD on AVT** | **p-value** |
| Decrease in LSM stage | 51.8% | 76.4% | <0.001 |
| Increase in LSM stage | 24.0% | 13.1% | 0.024 |

**Supplementary table 6. Association between MASLD and first LSM using multivariable linear regression based on higher cut-off CAP of >275 dB/m.**

|  | **Coefficient** | **p-value** |
| --- | --- | --- |
| **Age, years** | 0.075 | <0.001 |
| **Male sex** | 0.987 | <0.001 |
| **ALT, U/L** | 0.034 | <0.001 |
| **HBV DNA log_10_, IU/mL** | 0.116 | 0.030 |
| **Presence of MASLD** | 0.869 | 0.002 |

**Supplementary table 7. Association between MASLD and last LSM using multivariable linear regression based on higher cut-off CAP of >275 dB/m.**

|  | **Coefficient** | **p-value** |
| --- | --- | --- |
| **Age, years** | 0.021 | 0.044 |
| **Male sex** | 0.241 | 0.361 |
| **ALT, U/L** | 0.014 | <0.001 |
| **Use of antiviral therapy** | 0.070 | 0.804 |
| **First LSM, kPa** | 0.462 | <0.001 |
| **Time between first and last LSM, years** | -0.115 | 0.003 |
| **Presence of MASLD** | 0.709 | 0.021 |

**Table 8.** **Association between MASLD and risk of last LSM >9 kPa using multivariable logistic regression analysis based on higher cut-off CAP of >275 dB/m.**

|  | **aOR** | **95% CI** | **p-value** |
| --- | --- | --- | --- |
| **Age, years** | 1.034 | 1.012 – 1.056 | 0.002 |
| **Male sex** | 1.436 | 0.773 – 2.665 | 0.252 |
| **ALT, U/L** | 1.007 | 1.003 – 1.010 | <0.001 |
| **Use of antiviral therapy** | 1.501 | 0.816 – 2.761 | 0.192 |
| **First LSM, kPa** | 1.258 | 1.187 – 1.334 | <0.001 |
| **Time between first and last LSM, years** | 0.865 | 0.788 – 0.950 | 0.002 |
| **Presence of MASLD** | 2.648 | 1.512 – 4.635 | <0.001 |
